# The Experiences of Nurses in Care Provision to COVID-19 Patients: A Qualitative Study

**DOI:** 10.3389/fpubh.2022.766880

**Published:** 2022-04-29

**Authors:** Razieh Mokhtari, Ameneh Yaghoobzadeh, Kamel Abdi, Mahbobeh Sajadi, Mitra Jaras, Mohamad Golitaleb

**Affiliations:** ^1^Department of Nursing, School of Nursing, Arak University of Medical Sciences, Arak, Iran; ^2^Nursing Department, Faculty of Medicine, Komar University of Science and Technology, Sulimaniya, Iraq; ^3^Department of Nursing, Shazand School of Nursing, Arak University of Medical Sciences, Arak, Iran

**Keywords:** qualitative study, COVID-19, nursing, patients, pandemic (COVID19)

## Abstract

**Introduction:**

Nurses are key fighters in the forefront of care provision to COVID-19 patients. Due to the diversity of nurses' experiences in different countries because of variable nursing resources, health care systems, and cultural contexts, the present study aimed to divulge a deep understanding of the essence of health system problems based on nurses' experiences of care provision to COVID-19 patients in Iran.

**Methods:**

The present study was conducted based on the conventional content analysis method and Graneheim & Lundman approach. The participants included the nurses working in the COVID-19 wards and were recruited by purposeful sampling and based on inclusion criteria. The data were collected by conducting semi-structured, one-to-one interviews, and taking field notes, until reaching data saturation.

**Results:**

In-depth interviews with 12 nurses. represented four main categories and six subcategories. Sudden exposure to an unknown threat (nurses' feelings and concerns and nurses' reactions), being involved in an unequal war (a vicious virus and weary nurses), stressful working conditions, and efforts to confine the threat (seeking for new and adequate information and gathering all forces) were among the emerged data.

**Conclusion:**

The nurses' experiences showed that despite passing a while since the coronavirus pandemic, there are still individual and professional concerns that all root in organizational and governmental factors.

## Introduction

Nurses are key players at the forefront of providing care to COVID-19 patients, and their coordinated efforts are essential to put an end to the spread of the disease (1, 2). During the COVID-19 pandemic, nurses should be equipped with special skills to provide quality care to the patients who need their expertise, knowledge, attitudes, and skills, as well as their supportive care. So, health care workers should be skilled and accurate to be able to treat patients, and if nurses, as pioneers, lack the required expertise in inpatient care, they will impose major challenges on the health system (3, 4).

The rapid spread and high mortality of the COVID-19 disease have caused not only the public but also health care providers, especially nurses who are in close contact with infected people, the fear and anxiety about the impacts of the virus on themselves and their families (5, 6). In fact, nurses have expressed their great fear of either themselves or their family members being infected with the virus, and due to this risk, many of them are reluctant to work during the pandemic (7). Therefore, it is important to identify the complications and consequences of the pandemic on nurses and recognize the worries and concerns that can accentuate these problems (8). Various studies have been performed on the care provided by nurses to COVID-19 patients, noting that ethical issues and the lack of adequate information about emerging diseases (9) can significantly affect the health status of nurses and the quality of the care provided by them. In this regard, two studies showed that the nurses caring for patients with a new infectious disease such as SARS and H1N1 lacked precise information and instructions on how to provide patient care and utilize personal protective equipment (10, 11). Moreover, post-traumatic stress after witnessing the death of patients was another experience reported by the nurses providing care to patients with emerging respiratory infections (12–14). If these psychological problems are not effectively addressed, they may not only weaken nurses' immunity, which increases the risk of the COVID-19 infection, but may adversely affect the quality and safety of the health care system (15).

Due to variable nursing resources, differences in the structure of health care systems, and various cultural backgrounds, the experiences of nurses in various countries vary in terms of care provision to COVID-19 patients (5). The International Council of Nurses has recognized the key role of nurses in the treatment and care of patients with COVID-19 (16). Therefore, it seems necessary to acquire a deep understanding of nurses' experiences to establish a safe and efficient network in which health staff can be prepared for facing possible outbreaks of new infectious diseases in the future. Moreover, facing such a crisis and life-threatening conditions make patients to be completely or partially depend on the nurses who have to provide a physical needs, and their psychosocial (wellbeing and mental health) needs (17, 18). Also, some studies have shown that there some institutional and personal barriers that have determinant role in providing the suitable care for these kinds of patients (19, 20). Furthermore, our cultural views and morals about health/illness/treatment, and those of our patients, may not bring into line. Nurses must find shared ground in order to offer culturally sensitive care. During this pandemic we can make a difference by considering chances and tools to alleviate and lessen hidden prejudice. In addition to providing quality healthcare, we can accept our patient's cultural opinions related to health and illness and incorporate this information into the plan of care (21, 22). So, it seems to be important to care for patients' cultural belief and values in every stages of their treatment. To obtain a deep understanding of a certain phenomenon, it is required to perform qualitative research that makes it possible for decision makers to become aware of the phenomenon by knowing stakeholders' perceptions and insights and the factors that affect their performance (5). Despite the key role of nurses and their experiences on the quality of care, this issue has been neglected in Iran amid the COVID-19 pandemic. Due to the lack of studies on the experiences of nurses on care provision to COVID-19 patients, this study aimed to scrutinize an in-depth understanding of the essence of the health system's problems experienced by the nurses involved in care provision to these patients using a conventional content analysis approach.

## Methods

A qualitative content analysis approach was adopted for this study. Qualitative content analysis has been described as a “systematic and objective means of describing and quantifying phenomena” [(23), p. 1] Content analysis involves reducing data to concepts that describe a phenomenon like care provision. By creating “categories, concepts, a model, conceptual system, or conceptual map” [(23), p. 2], content analysis has been shown to help clarify and explain a given phenomenon because it reveals in-depth information about the participants' views.

### Participants

The participants in this study were 12 individuals with the mean age of 28.58 ± 3.9. To be included, participants needed to be working in the hospitals affiliated with Arak University of Medical Sciences, willingness to participate in the study, ability to communicate properly to convey rich and complete information, and having a bachelor's degree. The participants were therefore selected using a purposeful and criterion-based sampling approach. It has been argued that a sample size of between ten and twenty is appropriate for qualitative studies of this kind because they allow the researchers to discuss a sufficient breadth of responses in the appropriate depth (24). This sample size was also considered to be appropriate as the data analysis reached the point of saturation (25). All of the participants were from Arak city, Arak. They consented to taking part in the study verbally, and also using written consent forms.

### Data Collection

After the approval of the research protocol by the Ethics Committee of the School of Nursing, the researcher started to collect and analyze the data. Initially, participants were recruited and explained about the aims of the study, and then were requested to sign an informed consent form. Once the participants gave their consent to take part in the study, physical face-to-face interviews were arranged which took place in the participants' wards that they worked or the place that they felt comfortable, but generally 12 interviews were done during the shifts of the participants and four of them were done before or after the shift in the participants' rest room in the units that they worked. It needs to mention that no one was allowed to come to the room during the interviewing. The whole interviews were conducted by one of the researchers (MJ) with training in interview procedures and each interview was checked by MS. Semi-structured interviews were the preferred method of data collection because they offered the researchers flexibility to pursue, probe and clarify responses as they occurred, but also to make comparisons between participants. Sandelowski (26) purports that one-to-one interviews are the most commonly used data collection tools in qualitative research. Specifically, the authors used the one-to-one Semi-structured interviews due to the following reasons: (1) it is appreciated method of collecting rich in-depth data about participants' experiences and outlooks; (2) it suggests the researcher the chance to understand non-verbal indications through observation of body language, facial expression and eye contact and therefore may be seen to improve the interviewers consideration of what is being said; (3) it allows the researcher to investigate and discover unseen meanings and understanding; and (4) it provides valuable evidence about the public situation in which people exist (27).

Before starting the interview, the researcher aimed to build a rapport with the participants (28). Thereafter, the aims of the study were repeated. Participants were also informed that their responses would be confidential, and the process of recording the interview was also outlined. Data were grouped to ensure anonymity and confidentiality. Moreover, the author dedicated a number as a code to each participant in order to assure the confidentiality. After obtaining written consent, the interviewer began the interview using open questions.

Initially, four unstructured interviews were conducted to recognize the relevant questions needed to be asked, and then semi-structured interviews were held to gather information on the participants' positive and negative experiences and their opinions about priorities, strategies, and procedures in caring for COVID-19 patients.

In order to obtain maximum information, the highest diversity was tried to be fulfilled by recruiting nurses with variable working experiences (long and short) from different shifts (morning, evening, and night) and wards (intensive care units and general, etc.). An open question was initially asked, such as “What are your experiences in caring for COVID-19 patients?”. Other questions were asked based on the interview guide and the responses provided by the participants, including “What are your suggestions for caring for COVID-19 patients?”, “What are the impacts of the disease on your professional life and personality?”, and other similar questions. Based on the answers to these questions, follow-up questions were asked to explore the participants' responses. Examples of follow-up questions included: “What did you mean by this?” and “Can you explain this in more detail?” The duration of the interviews varied depending on the participants' responses and willingness to continue. Interviews lasted for approximately 20 to 70 minutes, with a mean interview length of 45 minutes. In all, 16 interviews were carried out with 12 participants. However, four interviews (No. 2, 4, 7, and 11) were repeated in order to clarify information from the first interview. After listening to the interviews' voices over and over for several times, their texts were transcribed verbatim on paper and then analyzed. All of the interviews were recorded with a digital voice recorder, conducted in Persian and translated to English. In order to record observations and the events and interactions occurring in the field, the researcher took field notes whenever necessary, which was guided by one of the researchers (MJ).

After collecting the required data using interviews, they were accurately analyzed using the conventional content analysis method based on the Graneheim & Lundman approach, which included five steps as follows:

1. Transcription of the Entire Interview Immediately After Its Conductance;
2. Reading the Entire Text to Acquire a General Understanding of Its Content;
3. Extracting the Units of Meaning and the Initial Codes;
4. Classifying Similar Primary Codes Into More Comprehensive Categories;
5. Determining the Main Themes of the Categories (29).

Having transcribed the data, the text was reviewed by one researcher, and notes were made based on her first impressions. As this process continued, the researcher began to identify code labels which reflected a wider range of the participants' thoughts and ideas. These codes made up the initial coding scheme, and often came directly from the text. Codes that were conceptually similar were placed in one cluster, and these semantically related clusters were then organized into themes. To ensure the reliability of the data analysis, two additional researchers reviewed the established codes and themes to ensure that they were an accurate reflection of the data. A fourth researcher was introduced to resolve disagreements and opposing interpretations among the first three researchers. A final version of the coding scheme was then agreed upon by all four researchers. The interviews continued until saturation of the data. Saturation in this study meant that no new code was created in the coding process and the generated codes were duplicates. Data saturation in the present study was obtained from the tenth interview and two other interviews were conducted to ensure the adequacy of sampling. After the sixteen interviews, all subsequent data could be coded using the final coding scheme.

## Data Accuracy and Rigor

Lincoln and Guba's method was also used in the current study. The assessed items of this method were as follows: credibility, dependability, transferability, and confirmability (30, 31). To assess the validity of the research, a trusted relationship was established with the participants. Each interview was provided to the participants after analyzing it, and their comments were sought to settle the data. Also, quotes reported were recorded verbatim. This study was conducted seeking expert colleagues' opinions on the extracted codes and categories for possible modifications. Moreover, the reviewers' suggestions were used throughout the research process. An external audit was used to assess the trustworthiness of the study. Finally, the audit process attested to the dependability of the study from a methodological standpoint, and the confirmability of the study by reviewing the data, analysis and interpretations, and assessing whether or not the findings accurately show the data. In essence, the audit observes both the process and product of the survey to control its trustworthiness. In this study, other advisors/supervisors and evaluators who were experts in qualitative research evaluate each phase of the research and provided ideas as needed.

One of the important subjects in qualitative research is the role of the researcher in eliciting data. The researcher as an instrument suggests opportunity to understand and discover an individual's experiences and insights of the phenomena in question. In order to suitably conduct qualitative research, the researcher should have the necessary experience and skills, and the ability to communicate (23).

## Results

Twelve nurses working in teaching hospitals affiliated with Arak University of Medical Sciences (Valiasr, Amir Al-Momenin, and Ayatollah Khansari) participated in this study and were subjected to in-depth interviews. The mean age of the participants was 28.58 (SD: 3.98) years, and the mean work experience was 5.5 (SD: 2.89) years. Most of the participants were married (66.6%). The participants' demographic information has been provided in Table 1.

**Table 1:** Demographic information of the nurses participating in the study.

**Demographic variables**		**Number (*n*)**	**Percentage (%)**
Gender	Female	11	91.66
	Male	1	8.33
Marital status	Married	8	66.66
	Single	4	33.33
Employment	Permanent	3	25
	Temporary to permanent	4	33.33
	Contract	5	41.66
Age (year), mean ± SD		28.58 ± 3.98	
Work experience (year), mean ± SD		5.25 ± 2.89	

The experiences of the participants in this study are presented in four main categories: sudden exposure to an unknown threat, being involved in an unequal war, stressful working conditions, and efforts to confine the threat.

### Sudden Exposure to an Unknown Threat

The nurses participating in this study clarified that they were encountered with the disease suddenly, a disease that was unknown, had a rapid spreading rate, affecting people's lungs and causing serious and even life-threatening respiratory problems. The disease also would rapidly infect other family members. In addition to these problems, there was insufficient information about its symptoms and transmission ways, as well as its preventive and therapeutic measures. The nurses suddenly encountered this problem without prior preparedness, which changed their routine work rapidly. This type of confrontation caused panic, fear, and shock in many of them. Data analysis revealed two subcategories of nurses' feelings and concerns and nurses' reactions to the COVID-19 disease.

#### Nurses' Feelings and Concerns

The data showed that many nurses experienced feelings such as incompetency, inefficiency, sadness, grief, unhappiness, indecision, inability to make decisions, fear of becoming infected and transmitting the disease to family members, and stress, anxiety, and worry about the complications of the disease or even their possible death. A number of nurses had even thought of quitting the profession due to these feelings, tensions, and difficult conditions. In this regard, a nurse stated:

“My biggest concern was the transmission of the disease to my family. I feared what if I was a carrier and transmit it to my family. What if my mom and dad would be unable to cope with the disease, and I be the reason of their death?”. The nurse continued:

“The first few months were very difficult. Stress was at the highest level. My colleagues were becoming infected one after the other. Even one of them was hospitalized here and constantly had hypoxia and dyspnea. I really was afraid of becoming infected myself.” (Participant No. 4)

### Nurses' Reactions

The data showed that a number of nurses sometimes expressed reactions such as oversensitivity to the disease, obsessive-compulsive behaviors, anger, crying, self-absorption, and irritability. In this regard, a nurse mentioned:

“This was our first experience. I was on a shift with my colleague, and we were so worried. We did not know what would happen? We thought we would die soon. We went into a room, hugged each other and cried...”. Another participant noted: “Every day, after taking off our protective clothing, we would spray each other's bodies with alcohol all over from the head to the toe to disinfect”.

Another Nurse Expressed:

“One of my colleagues was becoming very irritable and was always angry. The other one developed an obsession saying that she, after taking her child from the kindergarten, would spray him all over, change all his clothes and wash them with bleach, but still was thinking that he was contaminated, leaving her desperate.” (Participant No. 1)

### Being Involved in an Unequal War

Most of the participants in this study believed that they were dragged into an unequal war and did not know when it was going to end. A war in which, on one front, it was the COVID causative virus that seemed to be strong and designed for invading lungs, and on the other side, a small number of weary nurses. The nurses believed that on one hand, people would cause the disease to spread and consequently an increase in the number of referrals to hospitals by not observing health protocols; and on the other hand, there was this ever-changing virus with its vicious nature and the lack of a definite treatment. This type of fighting left nurses exhausted and depleted of energy and strength without seeing a clear vision ahead.

### Stressful Working Condition

Nurses expressed that they were working in a stressful condition due to factors including the large number of hospitalized people, the bad behaviors of patients' companions, the presence of severely ill patients in the ward, constantly hearing coughing and seeing people struggling for their breath, patients' intense fear, anxiety, and begging and their sudden and rapid death, seeing some patients being abandoned in the hospital and not having companions due to the fear of contracting the infection, as well as difficult working conditions such as wearing protective clothes, hats, and several layers of gloves, and not being able to drink fluids and water during work shifts, in addition to some organizational shortages such as insufficient number of nurses. A nurse stated:

“We were dressed like astronauts, wearing face masks and other protective clothes. It was very hot in them, and I was very helpless. I was thirsty and would like to drink some water, but I couldn't. I was afraid of getting infected.” (Participant No. 8)

Seeing patients' deaths was heartbreaking, and this was addressed by a nurse as:

“Patients were becoming perished in front of our eyes very rapidly. It was heartbreaking. A woman came to the hospital on her own in the morning. She was fine, but when they took a CT scan, her lungs were completely white. During the night shift, she developed dyspnea and died.” (Participant No. 5)

One of the nurses, addressing issues such as the lack of a proper patient management policy, the confusion of officials, and the lack of an appropriate system for rewarding and encouraging nurses (such as appreciating committed nurses by appropriate methods, stated:

“Nobody pays attention to us nurses here. We have compact work shifts. Managers do not care for proper disease management. One day, Ayatollah Khansari hospital becomes the center of Coronavirus, and the next day, Amir Al-Momenin hospital. Committed nurses do not get promotions or rewards, and because of this, they lose their motivation.” (Participant No. 1)

### Efforts to Confine the Threat

Most of the participants reiterated the necessity of continuous efforts to control the disease and confine the virus. According to the participants, nurses would do their maximum effort to bring the patient to the best health condition. This is fulfilled by providing either direct care to the patient or via appropriately training the families of patients and individuals with milder symptoms. By keeping themselves up to date and seeking new knowledge about the disease, nurses not only boost their own awareness, but also can provide the best care to patients. In this regard, one of the participants highlighted:

“In the ward where I work, all the colleagues are working beyond their capacity and abilities and try to provide patients with the best care so that they can recover as soon as possible.” (Participant No. 10)

Another Participant Noted:

“Nursing is a very hard profession. Anyway, from the beginning when we chose this field, we knew that we might face such a situation. So, even now, when we are under tremendous pressure, we are doing our best and even sacrificing ourselves, trying to get back to normal.” (Participant No. 6)

## Discussion

This study aimed to investigate the experiences of the nurses providing care to COVID-19 patients in Iran. Our results were categorized into four main categories, which will be discussed in two main areas in the following sections (Table 2).

**Table 2:** The categories and subcategories extracted from the experiences of the nurses providing care to COVID-19 patients.

**Categories**	**Subcategories**	**Examples of codes**
Sudden exposure to an unknown threat	Nurses' feelings and concerns	Sadness and grief
		Incompetency and
	Nurses' reactions	Oversensitivity to the disease
		Irritability
Being involved in an unequal war	A vicious virus	A virus designed to invade lungs
		The vicious nature of the virus
	Weary nurses	Nurses' exhaustion
		Medical staff severe fatigue
Stressful working condition		High workload
		Patients' sudden and rapid death
		The lack of adequate nurses
Efforts to confine the threat	Seeking for new and adequate information	Efforts to disclose the truth about the disease
	Gathering all forces	Implementing all skills

### An Unknown Threat, Exaggerated Stress, and Efforts to Control the Disease

According to studies on COVID-19, this disease, as a pandemic, has caused a severe shock to the health care system of most countries around the world (32, 33). The nurses participating in this study perceived the COVID-19 disease as a life-threatening condition. In fact, epidemic diseases can have a significant psychological impact on nurses whose presence is necessary for providing health care services (34). According to previous studies, pandemic diseases exacerbate nurses' stress as they are faced with severe emotional, physical, and cognitive demands and must adapt to them (35, 36). In the frontline of care provision, nurses face pain, death, and moral dilemmas. In addition, the shortage of human resources and lack of equipment make their work even more exhausting due to imposing a high workload and exposing them to potentially health threatening conditions (37). Consistently, Koh et al. and Lam et al. stated that poor control on the patient's condition, incompetent management, and poor planning would increase nurses' burnout during epidemics (38, 39). Based on our results, the impacts of the COVID-19 disease on nurses' health status bring them fear and panic that can significantly accentuate the job burnout syndrome among them. Such a scenario means that nurses are faced with a significant increase in physical and psychological demands in their profession, and this occupational threat can affect their personal and professional perceptions of existing demands and resources (34). Considering the job burnout caused by the perceived stress due to a shortage in available resources, it is important to evaluate the direct impacts of this perceived fear and its modifiers on job burnout and its relationship with occupational demands and resources. It is important to note that social, cultural, intrinsic, extrinsic, and personal factors can influence nurses' experiences and professional decisions. The results of various studies have shown that in dealing with the COVID-19 pandemic, it is required to make hard ethical and clinical decisions, and these two parameters are essential entities in order to provide quality, fair, and patient-oriented care services and greatly control the risk of harm to patients (40, 41). Therefore, the decisions made in this uncertain situation can have significant short-term and mid-term impacts on patients, their families, and health care providers. Therefore, incorrect decisions at this critical time can seriously inflict patients with consequences that may be even more devastating than the disease itself.

### An Unequal War and the Role of Nurses

In the present study, facing a dreadful disease was likened to presence in a battlefield, reminiscing an unfair fight against an invincible enemy such as the COVID-19 disease. Seshadri et al. provided an example to draw this unequal battle as: “Our weapon in this war is stone while the enemy (COVID) is equipped with a gun” (42). Also, Perron and Gagnon (43) described nurses as “foot soldiers” who are sent to a war without proper equipment (or even with no equipment), sufficient information, and adequate human forces and physical resources, and even without adequate support and compensation. Other studies have also referred to nurses as “war heroes” (44, 45). Likewise, in the present study, the nurses used the same drawings to describe their experiences and transfer their emotions, as well as to describe the difficulties they have faced and the impacts of these stressful situations on their physical and emotional well-being. It is obvious that such conditions can have no positive effects in the long-term. In fact, although appreciation may be psychologically supportive, the long-term shortage of equipment and facilities will have negative psychological consequences on various aspects of nurses' personal and professional lives. A study noted that nurses should criticize only in the favor and interests of the state but not otherwise (43).

On the other hand, not observing health protocols by the public has led to the establishment of the disease and its victory in this battle, a notion that was also mentioned by the participants of the present study. Accordingly, a study in Iran stated that the biggest challenges in fighting against and controlling the COVID-19 disease from the perspectives of physicians and nurses were the general public not taking the disease seriously and quarantine regulations not being strictly implemented for contaminated cities (46). A number of combat strategies have been proposed by various studies, including the quarantine of cities and self-quarantine, implementing travel bans and controlling the entry and exit of cities, observing personal hygiene, the provision of adequate health and protective equipment, helping people with their primary needs and livelihood, identification of those suspected to have the disease, and providing sufficient medical staff (47–49). Therefore, proper policymaking and planning, adopting coherent strategies for crisis and information management, and boosting public awareness can be substantially helpful in controlling the disease and preventing its adverse consequences on the society and nurses.

Generally, the importance of cultural perceptions in times of crisis is highlighted. Moreover, cultural sensitivity during a pandemic doesn't sound like an accolade-winning idea (50); that means although cultural beliefs and values seem to be an important factor, it could be considered as an unimportant agent in the life-threatening conditions when the humans' health has the priority. In this regard, Foster (51) stated that “all of the efforts to maintain a culture of safety and prevent harm have a common denominator: They're dependent on the hands, hearts, and minds of the staff”. So, during the life-threatening conditions, nurses feel more responsible to provide the suitable care, but it can vary based on cultural outlooks. This can make difference between nations. Iran is a country with an Islamic culture and a healthcare system that is unique from other countries. Iranian healthcare system is managed by pillars supported by religious and cultural sights. In Iran, patient care standards are controlled by Iranian beliefs in Islamic moral and ethical. Therefore, nurses from different social and cultural bases have diverse ethical and religious knowledge which may impact their care that they provide to the patients (52, 53). So, due to the importance of the cultural belief and values effectiveness, the nurses' outlooks considered as the main agent in doing the research especially the qualitative ones which reflects the individuals' point of views.

## Conclusion

The experiences of the nurses participating in the present study showed that despite passing a while since the onset of the coronavirus pandemic, there are still individual and professional concerns that mainly root in organizational and governmental issues. In fact, establishing appropriate national and cultural contexts with an emphasis on maintaining public health not only improves community health and causes a better and more effective disease management process, but also greatly reduces the workload of health care workers, including nurses.

## Implications for Practice and Limitations

Tackling with serious conditions like that of the COVID-19 pandemic which is reflected as an international threat, personnel of the health care organizations, and specially nurses, face serious challenges. Yet, if crisis is managed properly by getting enough information about all the aspects, environmentally and individually, nurses are more able to adopt with the current situation.

Due to the characteristics of qualitative research, the sample size of this study was limited. Moreover, all participants may not have revealed all their experiences due to worries about possible consequences. However, an effort was made to handle this limitation as much as possible by assuring the participants of the confidentiality and anonymity of their information.

## Data Availability Statement

The original contributions presented in the study are included in the article/supplementary material, further inquiries can be directed to the corresponding author/s.

## Ethics Statement

The studies involving human participants were reviewed and approved by Arak University of Medical Sciences. The patients/participants provided their written informed consent to participate in this study.

## Author Contributions

RM conceptualized the study with support from MG, AY, MJ, and MS. RM, KA, MS, and MG extracted the data for this study. RM cleaned and analyzed the data with support from MS. MG, AY, and MJ wrote the first draft of the manuscript. All authors contributed to writing and finalizing the manuscript. All authors have read and agreed to the published version of the manuscript.

## Funding

The study was conducted according to the guidelines of the Declaration of Helsinki and approved by the COVID-19 Research Center (3624) and the Research Ethics Committee (IR.ARAKMU.REC.1399.063) of Arak University of Medical Sciences.

## Conflict of Interest

The authors declare that the research was conducted in the absence of any commercial or financial relationships that could be construed as a potential conflict of interest.

## Publisher's Note

All claims expressed in this article are solely those of the authors and do not necessarily represent those of their affiliated organizations, or those of the publisher, the editors and the reviewers. Any product that may be evaluated in this article, or claim that may be made by its manufacturer, is not guaranteed or endorsed by the publisher.
